# Autophagy and porcine circovirus infection: a mini review

**DOI:** 10.3389/fcimb.2025.1667956

**Published:** 2025-09-09

**Authors:** Xiaoyong Chen, Xi Chen, Ziding Yu

**Affiliations:** ^1^ Xingzhi College, Zhejiang Normal University, Jinhua, China; ^2^ College of Life Sciences, Zhejiang Normal University, Jinhua, China; ^3^ Wuhu Vocational Technical University, Wuhu, China; ^4^ Detection of Food-borne Pathogenic Microorganisms Engineering Research Center of Wuhu, Wuhu, China

**Keywords:** autophagy, porcine circovirus, virus-host interaction, viral replication, viral pathogenesis

## Abstract

Porcine circovirus (PCV), particularly PCV type 2 (PCV2), is a major pathogen driving porcine circovirus-associated diseases (PCVAD), causing significant economic losses in the swine industry. Accumulating evidence highlights autophagy as a critical host-pathogen interface during PCV infection. PCV2 activates autophagy through reactive oxygen species (ROS)-mediated signaling and metabolic regulators like the AMP-activated protein kinase (AMPK)/mechanistic target of rapamycin (mTOR) axis, creating a conducive environment for viral persistence. Concurrently, this virus exploits ubiquitin ligases to induce ubiquitination of cellular immune factors, promoting selective autophagy for immune evasion. Host factors, such as retinol-binding protein 4 (RBP4), act as restriction factors by counteracting viral strategies through autophagy modulation. Environmental stressors could exacerbate PCV2 pathogenesis by amplifying ROS-dependent autophagy, while interventions like taurine mitigate viral replication via ROS/AMPK/mTOR pathway inhibition. This mini-review synthesizes current understandings of PCV-autophagy crosstalk, emphasizing its critical role as a host vulnerability and therapeutic target. Understanding the intricate interplay between autophagy and PCV infection may unveil novel therapeutic targets, such as autophagy modulators, to mitigate viral replication and immune pathology.

## Introduction

**Figure 1:**
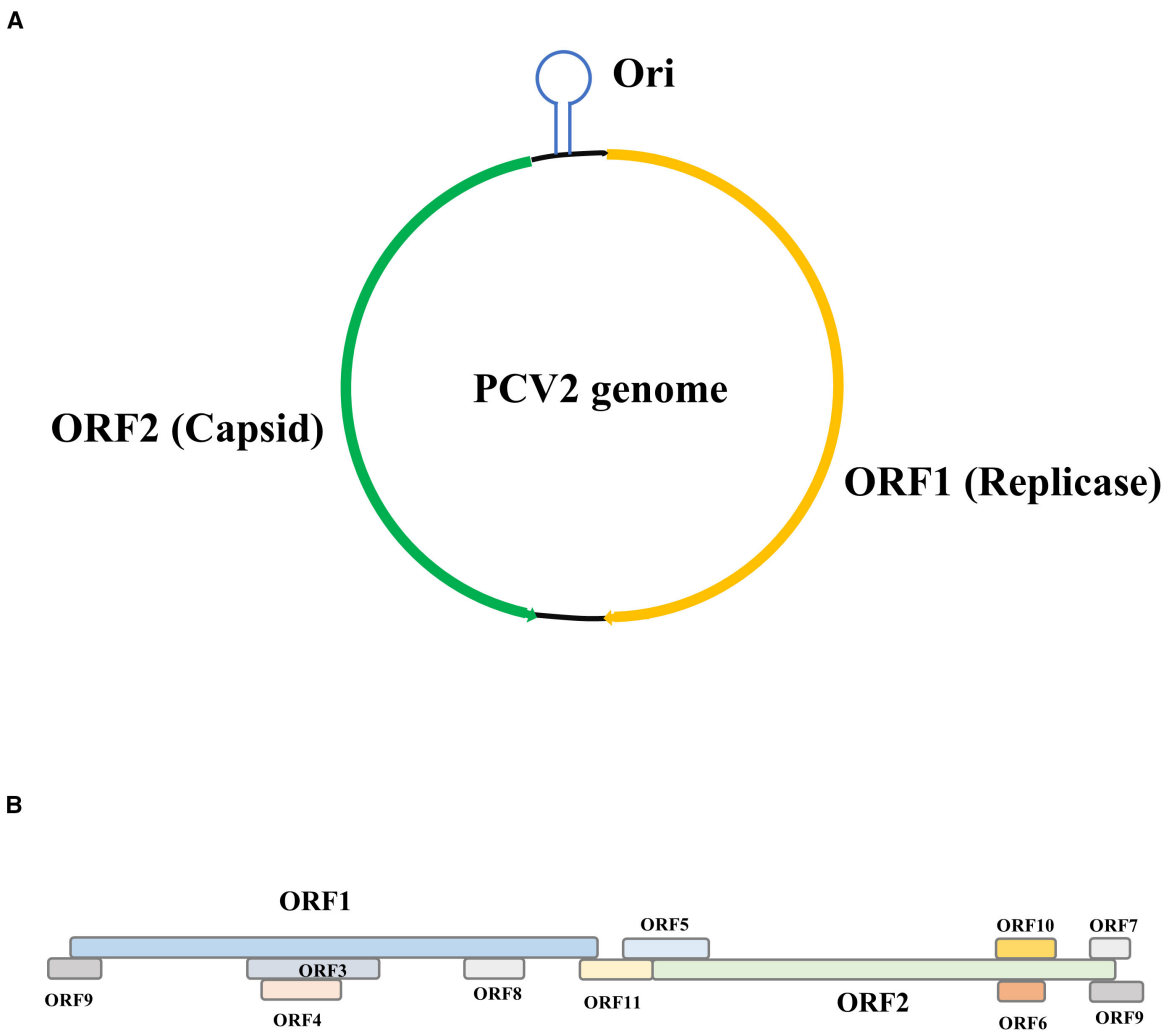
The structure of PCV2. **(A)** The genome of PCV2 is appropriately 1.7k nt. ORF1 is situated on the positive strand and predominantly encodes the Rep protein. In contrast, ORF2 resides on the complementary strand and is responsible for encoding the Cap protein. The intergenic region separating ORF1 and ORF2 contains the origin of replication (Ori), which is positioned between the initial codons of both ORFs. **(B)** The Depicts expanded open reading frames: ORF1 includes ORF9, ORF3, ORF4, ORF8, ORF11; ORF2 includes ORF5, ORF10, ORF6, ORF7, and ORF9.

Porcine circovirus (PCV), a member of the family *Circoviridae*, is a globally prevalent pathogen with significant economic impact on the swine industry (Opriessnig et al., 2020). First identified in 1974 as a non-pathogenic contaminant in porcine kidney cell cultures, its pathogenic potential emerged later with the recognition of postweaning multisystemic wasting syndrome (PMWS) in the 1990s, linked to PCV type 2 (PCV2) (Segalés et al., 2013). PCV is classified into four genotypes: PCV1 (non-pathogenic), PCV2 (pathogenic, associated with PMWS, respiratory disease, and reproductive failure), PCV3 (emerging, linked to reproductive disorders and multisystemic inflammation), and PCV4 (Opriessnig et al., 2020). Furthermore, PCV2 could be classified into six genotypes, including PCV2a-f (Franzo and Segalés, 2018; Mone et al., 2020). Structurally, PCV2 is a non-enveloped, icosahedral virus with a circular, single-stranded DNA genome (~1.7 nt), encoding two major proteins: the capsid (Cap) protein and the replicase protein (Rep) (**Figure 1**) (Rakibuzzaman and Ramamoorthy, 2021). The Cap protein forms the viral capsid, mediating host cell attachment and inducing neutralizing antibodies, while the Rep protein orchestrates viral DNA replication via rolling-circle amplification (Yan and Sun, 2024). Notably, PCV2 exhibits genetic diversity, with distinct genotypes differing in virulence and antigenicity. With a diameter of 17–20 nm, PCV is among the smallest autonomous animal viruses. Its small genome and minimalistic structure enable efficient replication in host cell nuclei, particularly in lymphoid tissues, where it disrupts immune homeostasis by targeting macrophages, dendritic cells, and lymphocytes (Fehér et al., 2023). The viral ability to modulate autophagy, apoptosis, and cytokine signaling underscores its intricate interplay with host defenses. Building upon the characterization of PCV2 pathogenesis and clinical manifestations, understanding PCV2 molecular architecture and evolutionary adaptations remains critical for developing vaccines and antiviral strategies against porcine circovirus-associated diseases (PCVAD). This knowledge not only elucidates viral persistence mechanisms but also informs rational design of therapeutic interventions targeting conserved viral epitopes or host-pathogen interaction nodes.

PCV infections, particularly those caused by PCV2 and PCV3, impose substantial economic and veterinary challenges globally. Clinically, PCV2 is strongly associated with PMWS, characterized by progressive weight loss, lymphoid depletion, and immune suppression, which predisposes pigs to secondary bacterial infections, exacerbating morbidity and mortality (Li et al., 2022). PCV3, an emerging pathogen, is linked to reproductive failure, including stillbirths, mummified fetuses, and neonatal mortality, as well as multisystemic inflammatory syndromes (Cobos et al., 2025). Affected pigs may exhibit nonspecific signs such as fever, lethargy, and diarrhea, complicating clinical diagnosis. Subclinical infections are also prevalent, leading to reduced growth rates and feed efficiency, which collectively diminish farm productivity. Pathologically, PCV2 induces granulomatous inflammation in lymphoid tissues, thymic atrophy, and interstitial pneumonia, while PCV3 is associated with vascular lesions and myocarditis (Li et al., 2025). Diagnosis relies on polymerase chain reaction (PCR), immunohistochemistry, or serological assays, though differential diagnosis is critical due to overlapping symptoms with other porcine diseases (Segalés, 2012; Goto et al., 2023). Prevention and control strategies include vaccination, which reduces viral shedding and clinical severity. Biosecurity measures, such as all-in/all-out systems, strict disinfection protocols, and minimizing stressors, are pivotal to limit viral transmission (Maity et al., 2023). Antimicrobial therapy may mitigate secondary infections, though antibiotic resistance concerns necessitate prudent use (Raith et al., 2016). Selective breeding for genetic resistance and nutritional optimization further support disease management (Chen et al., 2025). Emerging research on modulators, such as immune and autophagic factors, and antiviral peptides offers potential therapeutic avenues, underscoring the need for integrated, multidisciplinary approaches to combat PCV-associated losses.

Autophagy, a conserved cellular process involving the sequestration and lysosomal degradation of cytoplasmic components, plays an important role during viral infections, either inhibiting viral replication by eliminating viral particles or enhancing viral replication by evading immunity (He et al., 2024). For example, porcine epidemic diarrhea virus (PEDV) replication could be suppressed by a multitude of host factors via the autophagic degradation of nucleocapsid protein (Kong et al., 2020; Jiao et al., 2021). Conversely, porcine reproductive and respiratory syndrome virus (PRRSV) manipulates autophagy to establish persistent infections. The viral nonstructural protein 2 (nsp2) hijacks the autophagy machinery to degrade TANK-binding kinase 1 (TBK), suppressing activation of type I interferon (IFN) regulatory factor 3 (IRF3) and IFN-I production and enabling viral immune evasion (Zhao et al., 2024b). Additionally, classical swine fever virus (CSFV) exploits autophagy to inhibit apoptosis, ensuring viral persistence in host cells (Fan et al., 2021). Autophagy’s role in antigen presentation and cross-priming of T cells further complicates its relationship with viruses. While autophagy-derived viral peptides can enhance CD8+ T cell responses, some porcine viruses downregulate autophagy to avoid immune recognition (Wang et al., 2021a; Sun et al., 2022). Therapeutically, modulating autophagy—using agonists like rapamycin or inhibitors such as 3-methyladenine, shows promise in mitigating viral replication and immune pathology. However, tissue-specific and virus-specific context must be considered, as autophagic effects vary between viral species and infection stages. Thus, dissecting the intricate interplay between virus and autophagic processes is essential for designing precision therapies, as viral manipulation of autophagic pathways not only subverts host antiviral defenses but also creates actionable targets for pharmacological intervention.

The intricate relationship between autophagy and PCV underscores its significance as a research priority in virology and veterinary medicine. PCV2, the primary pathogenic type, induces autophagosome formation through interactions between its capsid protein and host autophagy-related proteins, facilitating viral genome release and capsid assembly in the nucleus. Studying this interplay is critical because autophagy modulation dictates infection outcomes: while excessive autophagy may promote viral persistence, its impairment exacerbates PCV-induced inflammation by accumulating damaged mitochondria and activating pro-inflammatory cytokines. Investigating autophagy not only elucidates PCV pathogenesis but also identifies therapeutic targets. Pharmacological agents that modulate autophagy could mitigate viral replication or hyperinflammation, offering innovative strategies to control PCVAD. Understanding this dynamic of PCV2 infection is thus pivotal for advancing swine health and reducing economic losses in the pork industry.

## PCV2 infection triggers autophagy via various pathways

**Figure 2:**
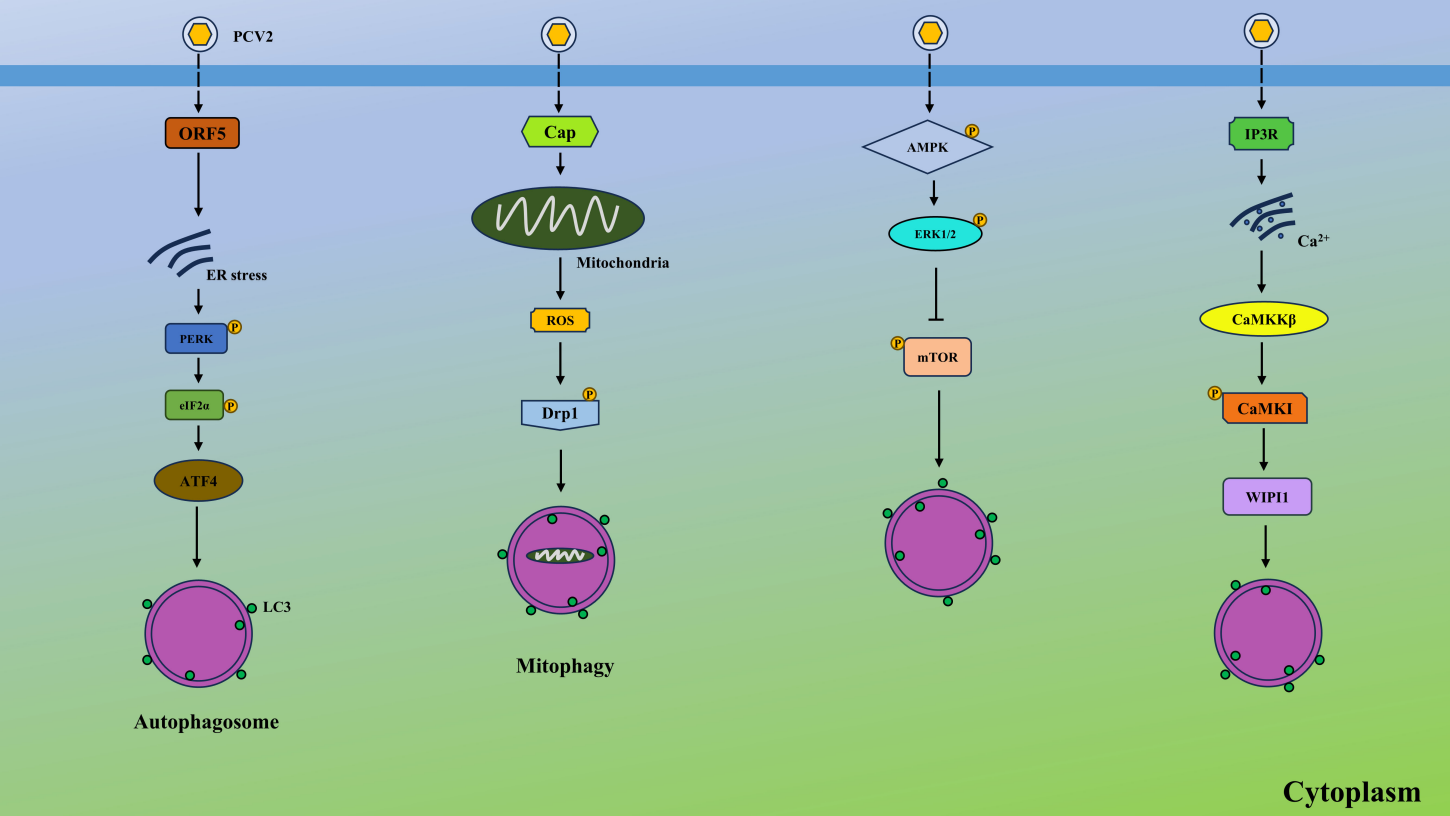
PCV2 induces autophagy via various pathways. PCV2 ORF5 causes ER stress, leading to autophagy via PERK-eIF2α-ATF4 axis. PCV2 Cap protein elevates the production of ROS from the mitochondrial and induces the mitophagy by the ROS-mediated activation of Drp1. In addition, PCV2 activates the AMPK and ERK1/2, suppressing the activation of mTOR and contributing to autophagy. Moreover, PCV2 activates the IP3R and elevates the cytosolic Ca^2+^ from ER, which upregulates CaMKKβ and activates CaMKI, finally generating WIPI1 and inducing autophagy.

PCV2 triggers autophagy through multiple interconnected pathways, forming a sophisticated network that regulates viral replication and pathogenesis (**Figure 2**). First, PCV2 induces endoplasmic reticulum stress (ERS), activating the PKR-like endoplasmic reticulum kinase (PERK)- eukaryotic initiation factor 2-alpha (eIF2α) arm of the unfolded protein response (UPR) (Lv et al., 2020). This leads to activating transcription factor 4 (ATF4)-mediated upregulation of pro-apoptotic proteins like Bcl-2, while simultaneously initiating autophagy via calcium (Ca^2+^) signaling. Elevated intracellular Ca^2+^, released through inositol trisphosphate receptor (IP3R) channels, activates calmodulin-dependent protein kinase kinase β (CaMKKβ), which bifurcates into two autophagy-inducing pathways: (1) CaMKKβ-AMP-activated protein kinase (AMPK) axis, where AMPK phosphorylates and inhibits mechanistic target of rapamycin complex 1 (mTORC1); and (2) CaMKKβ/CaM-kinase I (CaMKI)-dependent WD repeat domain, phosphoinositide-interacting 1 (WIPI1) recruitment, directly promoting autophagosome formation (Gu et al., 2016). Second, PCV2-induced mitochondrial dysfunction activates PINK1/Parkin-mediated mitophagy, clearing damaged mitochondria and reducing reactive oxygen species (ROS) accumulation (Zhang et al., 2020). Third, PCV2 induces autophagy via the AMPK/extracellular signal-regulated kinases 1 and 2 (ERK1/2)- tuberous sclerosis protein 2 (TSC2)-mTOR signaling axis. AMPK and ERK1/2 activate autophagy by inhibiting mTOR through TSC2 phosphorylation in infected cells (Zhu et al., 2012). Lastly, PCV2 could induce p38 phosphorylation and autophagy to exacerbate ochratoxin A-induced nephrotoxicity (Gan et al., 2018). Intriguingly, PCV3 was also reported to induce autophagy by its capsid protein in HEK293T cells, as shown by formation of autophagosomes and autophagosome-like vesicles via suppressing phosphorylation of the mTOR (Geng et al., 2020). Collectively, these pathways synergize to create a pro-autophagic environment that supports PCV persistence and pathogenesis.

## Autophagy promotes PCV2 replication

Increasing evidence showed that autophagy has a pro-viral role in enhancing PCV replication through multifaceted interactions between viral components and host autophagic machinery. For example, ochratoxin A (OTA), a mycotoxin produced by *Aspergillus* and *Penicillium*, enhances PCV2 replication by inducing autophagy in porcine kidney PK-15 cells. Autophagy inhibitors reduce OTA-driven PCV2 amplification. ROS scavengers block OTA-induced autophagy, suggesting ROS involvement. In pigs, OTA elevates PCV2 replication and autophagy in key tissues (Qian et al., 2017). Similarly, Zhai et al. found that oxidative stress could promote PCV2 replication via induction of autophagy (Zhai et al., 2019). Notably, interventions targeting autophagy, such as the use of autophagy inducers, have been shown to amplify PCV replication *in vitro*, underscoring the critical dependency of viral propagation on host autophagic flux. For instance, microRNA-30a-5p and miR-214–5p exert positive effects on PCV2 replication via various mechanisms. The former targets a 14-3–3 gene, which was a modulator of autophagy (Wang et al., 2017). The latter disrupts protein kinase B (PKB, also known as AKT)/mTOR signaling, thereby inducing autophagy and boosting viral replication (Cao et al., 2023). Additionally, a heat shock protein named DNAJB6 also serves to positively regulate PCV2 replication by interacting with Cap protein and promoting the production of autophagosome (Han et al., 2020). Interestingly, Liu et al. discovered that glutamine deficiency in host cells contributes to upregulated ROS-medicated Janus kinase 2 (JAK2)/signal transducer and activator of transcription 3 (STAT3) signaling and caused autophagy, thereby facilitating PCV2 replication (Liu et al., 2018a). Recently, porcine cGAS was found to become a target by PCV2 and degraded via autophagy in PCV2-infected cells, impairing cGAS-STING signaling and aiding in viral replication (Wang et al., 2021b). These findings position autophagy as a central node in PCV pathogenesis, with therapeutic implications for developing antiviral strategies by modulating autophagic processes.

## Targeting autophagy impedes PCV2 infection

A multitude of studies indicate the positive role of autophagy in PCV2 replication. Based on this, pharmacological inhibitors and additions could be used to effectively suppress PCV2 proliferation by disrupting autophagic flux, as exhibited by reduced viral titers, impaired capsid protein expression, and decreased DNA copy numbers.

## Antioxidants and metabolic regulators

Antioxidants and metabolic regulators play important roles in modulating autophagy. For example, ROS could act as signaling molecules that induce autophagy under oxidative stress, while antioxidants like N-acetylcysteine (NAC) may attenuate excessive autophagy by reducing ROS levels (Qi et al., 2024). Metabolic regulators, such as AMPK and mTOR form a regulatory axis, where AMPK activates autophagy during energy deprivation, and mTOR inhibits it under nutrient-rich conditions (Kim et al., 2011). Studies have shown that compounds like metformin (AMPK activator) or rapamycin (mTOR inhibitor) demonstrate how metabolic interventions can fine-tune autophagic flux (Bharath et al., 2020).

Taurine, known as a sulfur amino acid regulated by the kidney, could attenuate ROS level and block OTA-mediated autophagy, thus impairing PCV2 replication. Mechanistically, taurine modulates the ROS/AMPK/mTOR signaling axis by inhibiting AMPK and activating mTOR, while chemical AMPK activation via acadesine (AICAR) abrogated taurine’s antiviral activity (Zhai et al., 2018). Similarly, SeMet, the major component of organic selenium, significantly inhibits OTA-induced enhancement of PCV2 replication. Furthermore, SeMet attenuated OTA-triggered autophagy and reverses the OTA-mediated suppression of p-AKT and p-mTOR expression (Qian et al., 2018). Rapamycin, an AKT/mTOR inhibitor, abrogates SeMet’s suppressive effects on both OTA-induced autophagy and PCV2 replication enhancement. Above studies indicate these regulators hold therapeutic potential to dysregulated autophagy during PCV infections, offering targets for pharmacological intervention through antioxidant supplementation or metabolic pathway modulation.

## Natural product-based autophagy modulators

Natural product-derived autophagy modulators represent a promising class of bioactive compounds that finely tune autophagic processes through diverse molecular mechanisms. For instance, resveratrol activates autophagy by inhibiting the mTOR pathway or enhancing AMPK signaling, while flavonoids such as quercetin may suppress excessive autophagy under oxidative stress via ROS scavenging (Tang et al., 2020; Xia et al., 2024). Marine-derived metabolites, including terpenoids and alkaloids, often target Beclin-1 or ATG proteins to regulate autophagosome formation (Jiang et al., 2019; El-Baba et al., 2021). Notably, these modulators exhibit context-dependent dual roles, either promoting protective autophagy in diseases or inhibiting pathological autophagy. Paeonifforin was demonstrated to disrupt AKT/mTOR signaling, thus suppressing autophagy, which in turn impeded PCV2 replication (Wu et al., 2025). Another study found that astragalus polysaccharide (APS) and selenizing APS (sAPS) could provide protection against PCV2 infection (Liu et al., 2018b). In detail, they activated phosphatidylinositol-3-kinase (PI3K)/AKT signaling and downregulated autophagy, contributing to decreased PCV2 replication. These findings suggest that targeting autophagy using natural products may offer a novel antiviral strategy against PCV-associated diseases.

## Conclusion and perspectives

The intricate interplay between autophagy and PCV infection has emerged as a pivotal axis in understanding viral pathogenesis and host immune modulation. Autophagy plays a role by degrading viral components and modulating immune responses during PCV2 infection. While PCV2 has evolved sophisticated strategies to manipulate autophagy for their replication, persistence, and immune evasion. However, evidence of autophagy regulating PCV3 and PCV4 infections is lacking and needs further investigations in the future. PCV2 infection elevates intracellular ROS levels, which in turn induces autophagosome formation (Zhang et al., 2020). Simultaneously, viral proteins such as ORF1 interact with host ubiquitin ligases like TRAF6, promoting K63-linked ubiquitination of viral components to facilitate their recognition by autophagy receptors like SQSTM1/p62 (Han et al., 2024). This selective autophagy ensures viral protein degradation is suppressed until replication peaks, after which autophagic flux is redirected to degrade antiviral host factors. Notably, PCV2 upregulates adipokine RBP4, which amplifies TRAF6-dependent ubiquitination of viral proteins, creating a feedforward loop that sustains autophagy activation. Paradoxically, excessive autophagy induced by viral manipulation triggers lysosomal dysfunction, enabling viral particles to escape degradation (Zhao et al., 2024a). This modulation of autophagy—promoting its initiation while inhibiting late-stage maturation—creates a niche for persistent infection. Furthermore, environmental factors like ochratoxin A (OTA) synergize with viral strategies by enhancing ROS-dependent autophagy, whereas dietary antioxidants such as taurine counteract this process through AMPK/mTOR pathway modulation (Qian et al., 2017). These findings underscore PCV2’s evolutionary adaptation to co-opt autophagy, highlighting the intricate balance between viral pathogenesis and host defense mechanisms.

Pharmacological interventions targeting autophagy pathways hold promise for controlling PCV infection. Autophagy inhibitors, such as 3-methyladenine (3-MA) and chloroquine (CQ), could theoretically limit viral replication by blocking autophagosome formation or lysosomal degradation. CQ disrupts lysosomal acidification, thereby preventing viral capsid disassembly and genome release. Conversely, autophagy inducers like rapamycin may enhance antiviral immunity by promoting autophagic clearance of viral particles and dampening excessive inflammation. For example, retinol-binding protein 4 (RBP4), an adipokine and retinol carrier, triggers autophagic degradation of the viral ORF1 protein through K63-linked ubiquitination, which recruits SQSTM1/p62 for delivery and degradation, thus reducing viral replication and impairing its pathogenicity (Han et al., 2024). Moreover, Matrine, a quinolizidine alkaloid, suppresses PCV2 infection, protects the intestinal barrier function, and promotes intestinal clearance of virus in murine models by activating cellular autophagy (Wang et al., 2024). These studies indicate that autophagy could be a promising therapeutic target to regulate PCV infection.

Emerging research highlights specific viral proteins as therapeutic targets. The PCV2 Cap protein interacts with host ATG proteins to hijack autophagy. Disrupting this interaction using peptide inhibitors or monoclonal antibodies could selectively impair viral replication without compromising global autophagy. Similarly, targeting PCV-induced autophagy-related membrane trafficking may offer genotype-specific interventions. Traditional Chinese medicine (TCM) compounds with immunomodulatory and antiviral properties may provide novel autophagy-based therapies. For example, berberine, isolated from Coptis chinensis, induces autophagy-dependent clearance of intracellular pathogens by activating various signalings (Mohammadinejad et al., 2019). Paeonifforin inhibits PCV2 replication by blocking autophagy via disturbing AKT/mTOR signaling (Wu et al., 2025). Glycyrrhizin from Glycyrrhiza uralensis alleviates anti-inflammatory effects by modulating autophagy via the PI3K/AKT/mTOR pathway (Qu et al., 2019). High-throughput screening of TCM libraries may identify additional candidates that restore autophagic flux or counteract viral subversion.

Autophagy represents a critical nexus in PCV pathogenesis, offering both a vulnerability to exploit for therapeutic intervention and a mechanism of viral resilience. Advances in understanding the spatiotemporal dynamics of autophagy during infection, coupled with innovations in drug delivery and TCM-derived compounds, position autophagy modulation as a cornerstone of future antiviral strategies. Integrating omics technologies, clustered regularly interspaced short palindromic repeats/CRISPR-associated protein 9 (CRISPR-Cas9) screens, and artificial intelligence (AI)-driven drug repurposing will accelerate the translation of these insights into clinical solutions, ultimately reducing the global burden of PCV-associated diseases in swine and safeguarding food security.
